# A Cross-Sectional Study of Individual Learning Passion in Medical Education: Understanding Self-Development in Positive Psychology

**DOI:** 10.3389/fpsyg.2022.758002

**Published:** 2022-03-17

**Authors:** Shu-e Zhang, Si-ao Ge, Jing Tian, Qing-lin Li, Ming-si Wang, Xiao-he Wang, Meng Zhang, Ji-yang Zhao, Li-bin Yang, De-pin Cao, Tao Sun

**Affiliations:** ^1^Department of Health Management, School of Health Management, Harbin Medical University, Harbin, China; ^2^Office of Academic Research, Harbin Medical University, Harbin, China; ^3^Department of Health Management to School of Medicine, Hang Zhou Normal University, Hangzhou, China; ^4^Department of Education, Harbin Medical University, Harbin, China; ^5^Center for Higher Education Research and Teaching Quality Evaluation, Harbin Medical University, Harbin, China; ^6^Department of Health Management, School of Medicine, Hangzhou Normal University, Hangzhou, China

**Keywords:** medical students, learning passion, self-esteem, psychological capital, professional identity

## Abstract

**Background:**

Boosting the individual learning passion of medical students is a novel approach to improve their academic performance. It facilitates the medical education reform, motivating both policymakers and educators to focus on the function of positive psychology in the career development of medical students. Therefore, this study aimed (1) to assess the status of two types of learning passion; (2) to clarify the relationship between self-esteem and two types of learning passion among Chinese medical students; (3) to examine the mediating role of psychological capital (PsyCap) in the relationship between self-esteem and two types of learning passion, respectively; and (4) to identify the moderating role of professional identity in the relationship between PsyCap and two types of learning passion, respectively.

**Methods:**

A cross-sectional online survey was conducted from April to June 2016 in China. A total of 1,218 valid questionnaires (effective completion rate: 67.93%) were collected from four medical schools.

**Results:**

Self-esteem significantly and positively influenced medical students’ PsyCap (β = 0.637, *P* < 0.01) and two types of learning passion, including harmonious learning passion (β = 0.589, *P* < 0.01) and obsessive learning passion (β = 0.436, *P* < 0.01). PsyCap fully mediated the relationship (β = 0.578, *P* < 0.01) between self-esteem and harmonious learning passion positively, whereas it suppressed the relationship (β = 0.490, *P* < 0.01) between self-esteem and obsessive learning passion. Further, professional identity significantly moderated the correlation between PsyCap and harmonious learning passion (β = −0.554, *P* < 0.05), rather than obsessive learning passion (*P >* 0.05).

**Conclusion:**

Two types of learning passion of medical students are positively influenced by self-esteem and PsyCap. Medical students with high-level self-esteem should possess strong PsyCap, which augments their harmonious as well as obsessive learning passion. Moreover, the positive effect of medical students’ PsyCap on harmonious learning passion is more notable among those with a lower professional identity. Finally, this study argues that strengths-based interventions of self-esteem and PsyCap are a beneficial approach for future enhancing learning passion in the domain of medical education.

## Introduction

Currently, a low degree of professional commitment and a high level of learning burnout among medical students in China have become noticeable issues in need of an urgent solution (Jinghua et al., 2014; Xie et al., 2019). Positive education has proved to be an emerging paradigm by introducing positive psychology principles into the domain of education. This aims to promote students’ wellbeing and academic excellence (Julie, 2021; Morgan and Simmons, 2021). Passion for learning is an important concept that stems from positive education. Learning passion seems to provide new understanding approach of improving the academic performance and academic wellbeing, as well reduced burnout among medical students (Chen et al., 2021). Medical students’ learning passion could directly contribute to proficiency in medical knowledge and skills and enhance the potential quality of future clinical work in health services (Rani et al., 2020). Moreover, the study on individual passion can trigger the positive characteristics of medical students, promoting beneficial outcomes in the domain of medical education (Brunzell et al., 2016), which seems to have been widely ignored. In addition, teachers and parents attached great importance to the education of Chinese students (Liu and Tein, 2005). Compared to general professional learners, medical students need to fully absorb learning materials from several courses and endure a long academic journey that involves professional and practical curricula (Abdulghani et al., 2012). This contributes to increasing academic pressure and anxiety and decreasing learning enthusiasm and learning engagement (Sun et al., 2017), which may significantly affect the academic performance and wellbeing of the students. Being a component of individuals’ psychological factors, passion for learning is beneficial in helping the students engage in an activity for a long time and exerting a remarkable influence on the student’s academic performance (Mageau et al., 2009), belongingness (Stenseng et al., 2015), and interpersonal relationships (Vallerand, 2008). Furthermore, harmonious learning passion has been significantly and positively correlated with individuals’ positive emotional experiences in school and a series of positive indexes of psychological adjustment (life satisfaction, vitality, etc.); However, obsessive learning passion has been significantly negatively correlated with the undesirable indexes, such as alcohol-related problems, and mental health (Vallerand et al., 2003, 2007). In addition, the personal characteristics and psychological states of medical students, such as self-esteem, optimism, psychological resilience, and other PsyCap, are crucial for the formation of optimistic and healthy psychological traits and the promotion of academic performances for future medical and clinical work. Currently, much of previous research regarding the influence of passion has been extensively discussed in different occupational groups; however, limited academic attention has been given to the research on learning passion in the domain of education (Yim et al., 2008; Oh, 2021; Violet et al., 2021), especially in the Chinese cultural background. In particular, studies on the learning passion of medical students and the factors affecting it are still needed.

### Dualistic Passion

In 2003, Robert J. Vallerand and his team summarized and defined passion as the strong will or emotional inclination of an individual to engage in an activity that they are attached to, and they are willing to invest a great deal of time and energy in Vallerand et al. (2003). Drawing from self-determination theory (SDT), scholars have developed a two-dimensional model of general passion by addressing a distinction between harmonious and obsessive passion (Vallerand et al., 2010). Harmonious passion originates from an autonomous internalization of individual activity into self-identity. Autonomous internalization occurs when a person willingly accepts the activity of one’s own accord with self-directed behaviors, following a sense of volition and personal endorsement. Moreover, harmonious passion can be coordinated with other aspects of an individual’s life, which can result in a series of positive outcomes. However, obsessive passion arises from the process of controlling the internalization of the activity. Although a person with obsessive passion tends to trigger a strong motivation to engage in activities, the prescribed course of internalized actions is more likely to be affected by external or environmental force, interpersonal or self-pressure, or interpersonal environments—self-esteem or social identity. This may further result in a strong sense of reluctant drive and control (Zylan, 2011). Moreover, obsessive passion has an overpowering effect, engendering potential conflicts with other activities in different aspects of one’s life, which tends to result in different outcomes for the individual, within a controllable range (Mageau et al., 2009). Individuals have a passionate tendency to assimilate and integrate external behavior (Deci and Ryan, 1985). Specifically, the Dualistic Model of Passion (Vallerand et al., 2003) has suggested that people can experience two types of passion toward an activity, further causing diverse affective, cognitive, and behavioral outcomes. Unfortunately, inadequate research has been conducted regarding how psychological elements independently affect one’s dualistic passions; therefore, this study aims to enrich the research on the issues mentioned above through an academic discussion. It is the first study to explore the association between self-esteem, psychological capital and two types of passions and the underlying mechanisms among Chinese medical students.

### Internal Relationships Between the Two Types of Passion, Self-Esteem, and Psychological Capital

**FIGURE 1:**
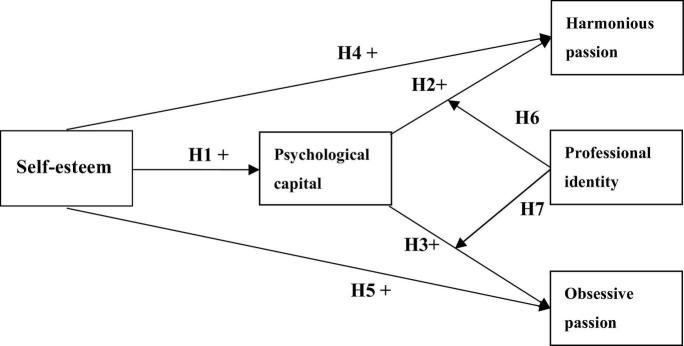
Hypothesis model.

The learning passion of medical students could directly affect their proficiency in medical knowledge and skills and potentially benefit the quality of future clinical work in health services (Rani et al., 2020). In this study, we propose a conceptualization of learning passion as a strong inclination toward learning activities that students would value and like, and in which they invest a substantial amount of time and energy. Driving passion for learning *activities* requires a series of psychological characteristics, states, or resources involving subjective experience, self-evaluation, psychological resources, self-identification, and self-psychological experience (Newell, 2003; Ivanova, 2021). Self-esteem, as a comprehensive positive or negative self-evaluation of a person’s self-worth, refers to the motivation of individuals to evaluate themselves positively and maintain this positive evaluation (Rosenberg, 1965a). A study indicated that higher self-esteem was likely to stimulate enhanced initiative and pleasant feelings and had been related to greater happiness (Baumeister et al., 2003). Psychological capital refers to one’s positive psychological resources—self-efficacy, hope, optimism, and resilience—which usually focuses on how an individual is changing or evolving rather than who that individual is (Luthans et al., 2007a). Previous results found that college students’ PsyCap had a significantly positive relationship with positive emotions, achievement motivation, learning empowerment, academic performance (Carmona-Halty et al., 2019), and wellbeing (Ji, 2016). According to a self-consistency revision of cognitive dissonance theory (Stone, 2003), we inferred that medical students with high self-esteem are less likely to succumb to feelings of incompetence and self-doubt and have greater aspirations. Thus, high self-esteem contributes to driving the process of passion for learning among medical students and establishing positive psychological resources for them. Owing to the existing conflict between obsessive passion and other activities, they are more likely to experience self-esteem fluctuations that covary with their performances in their passionate activities (Mageau et al., 2011). Previous study has found that obsessive passion could be driven by the need for social acceptance or self-esteem (Vallerand et al., 2003, 2007). Conversely, individuals with high-level harmonious passion do not experience self-esteem fluctuations (Mageau et al., 2011). Although there posing multiple challenges for researchers to measure passion and test passion-linked theories across different cultural contexts. However, it is possible that cultural differences could be driving the cultural variation in the link between the two types of passion and self-esteem, especially in China with the characteristic of collectivism (Li Q. L. et al., 2021). Therefore, we speculate that two distinct types of passion arise as a result of a continuous spiral internalization process that varies in the level of its development in the learning process (Vallerand et al., 2007). This is more likely to comprise a series of complex interactive relationships rather than the single directional relationship between the variables previously mentioned (Ji, 2016; Carmona-Halty et al., 2019); thus, we attempt to explore the following five research hypotheses in this study, hypothesis were shown Figure 1.

Hypothesis 1: Self-esteem is positively related to PsyCap.

Hypothesis 2: PsyCap is positively related to harmonious learning passion.

Hypothesis 3: PsyCap is positively related to obsessive learning passion.

Hypothesis 4: Self-esteem is positively related to harmonious learning passion.

Hypothesis 5: Self-esteem is positively related to obsessive learning passion.

#### The Moderating Effect of Professional Identity

The conceptualization of professional identity is the individuals’ sense of oneness regarding a profession (e.g., medicine) (Olesen, 2001) and a consistency degree to which they are willing to define themselves as professional members (Olesen, 2001). For medical students, professional identity tends to affect their understanding regarding the recognition of the medical profession and the degree of emotional connection with their major (Joseph et al., 2017). The self-identification of students with the medical profession has been linked with a successful transition from being a medical student to a professional doctor, in addition to a series of positive outcomes—PsyCap, learning efficiency (Li and Yang, 2018), achievement motivation, and learning satisfaction (Jianyu and Dan, 2016). Although evidence exists regarding the positive effect of activity identification on harmonious as well as obsessive passion (Mageau et al., 2009), some studies account for the multitude of relationships between PsyCap, professional identity, and learning passion. Previous studies found that identity played a moderator in the multiple relations between self-efficacy, self-concepts, self-feeling, motivation, and behavior-relevant outcomes (De Cremer, 2005; Lim and Kim, 2011). Professional identity is a subjective judgment and can be influenced by one’s psychological characteristics, behavioral norms, and extrinsic work values toward the medical discipline (Monrouxe, 2010; Joseph et al., 2017). Moreover, it represents the extent of the cognitive connection between the attributes and goals that subjects often identify themselves with. We inferred that PsyCap, interacting with different levels of professional identity, is more likely to drive different types of learning motivation during medical students’ learning process. Based on the above, we formed the following hypotheses:

Hypothesis 6: Professional identity moderates the relationship between PsyCap and harmonious learning passion.

Hypothesis 7: Professional identity moderates the relationship between PsyCap and obsessive learning passion.

#### The Mediating Role of Psychological Capital

PsyCap, as a cognitive-motivational variable, has been linked with several positive outcomes and plays a key role in the relationships between the various school-related factors (Luthans et al., 2007a; Siu et al., 2014; Kim and Shin, 2021; Lin, 2021). Similar research has found that positive study-related emotions could enhance students’ learning engagement, academic performance, and achievement (Villavicencio and Bernardo, 2013). Moreover, related evidence has indicated that individuals with positive beliefs about personal competence could respond by adaptively facing stressors, thereby predicting positive states of spiraling gains (e.g., learning passion and engagement) (Datu et al., 2018). Specifically, many studies concluded that both high-level PsyCap and self-esteem are positively related to better academic achievement, greater academic engagement and adaptation, and more positive learning attitudes (Datu et al., 2018; Carmona-Halty et al., 2019). Dualistic passion increases based on the SDT (Vallerand et al., 2003), involving harmonious and obsessive passion, and exerts prominently different processes of motivation internalization owing to distinct psychologically functional mechanisms (Vallerand et al., 2003). Thus, we can infer that students who have a stable and high level of self-esteem—that can inspire learning interest—tend to perceive more positive emotions during the learning period. This arouses learning motivation and awakens learning passion and engagement through different paths. The proposed mediational mechanism occurs because study-related positive emotions, such as self-esteem, may facilitate the construct of PsyCap and, in turn, foster two types of learning passion through increasing resource caravans. Therefore, PsyCap, as a cognitive-motivational factor, likely plays a mediating role in the relationship between self-esteem and the two types of passion. A study has revealed that PsyCap could positively predict two types of motivation—controlled and autonomous motivation. According to the study, students with PsyCap tend to achieve higher academic performance and exhibit greater adaptability of school-related activities (Datu et al., 2018). Thus, based on the above statements, we propose the following hypotheses:

Hypothesis 8: PsyCap mediates the relationship between self-esteem and harmonious learning passion.

Hypothesis 9: PsyCap mediates the relationship between self-esteem and obsessive learning passion.

### Objectives of the Study

The previous research highlights the following significant goals in our study: (1) to assess the status of two types of learning passion; (2) to clarify the relationship between self-esteem and two types of learning passion among Chinese medical students; (3) to examine the mediating role of PsyCap in the relationship between self-esteem and two types of learning passion, respectively; and (4) to identify the moderating role of professional identity in the relationship between PsyCap and two types of learning passion, respectively.

## Materials and Methods

### Subjects and Procedures

A combination of multistage stratified and convenient sampling was used in this study. For the convenient sampling process, an anonymous online questionnaire was completed by medical students from Chengde Medical College, Qiqihar Medical College, Mudanjiang Medical College, and Harbin Medical University, from April to June 2016. The participants who are different classes and grades were randomly selected in surveyed medical universities. First, we contacted the teachers in charge of student affairs and the academic administrators as the original deliverers of the survey. Subsequently, a webpage link to our self-administered questionnaire^1^ was sent by the teachers to the students via mobile phones. Each participant can respond only once. Furthermore, researchers use the Questionnaire Star platform to monitor the collected questionnaires in real time and manage the data effectively. Senior investigators conduct quality control by checking the collected questionnaires on a daily basis. A total of 1,793 students completed their questionnaires. For the purposes of data management and quality control, strict adherence to exclusion criteria was maintained. Finally, 1,218 valid questionnaires were collected, with an effective completion rate of 67.93%, excluding unanswered or incomplete questionnaires, those answered in an extremely short period, and/or those with an excessive number of blank items. The inclusion criteria were recognized as (1) medical students who is studying in a medical college; (2) voluntarily and truthfully cooperating with the online questionnaire survey; (3) complete answers. Similar survey method has been successfully used in international studies (Zhang et al., 2018; Li X. et al., 2021; Shi et al., 2021).

### Measurements

#### The Measurement of Learning Passion

Referring to the primitive General Passion Scale developed by Vallerand et al. (2003), a Learning Passion Scale was revised to adapt to the group of medical students (Ge et al., 2016). This primary tool had 12 items and was applied to medical students; this tool obtained high reliability and validity in a previous study conducted in China (Ge et al., 2016). In this study, after conducting a factor analysis, the remaining Medical Students’ Learning Passion Scale presented 12 items and was divided into two dimensions: the passion of harmonious learning and that of obsessive learning, with six items each. We used a seven-point Likert scale ranging from 1 (*completely inconsistent*) to 7 (*completely consistent*). The score equaled the sum of the average of each item, and a higher score presented a higher level of student’s learning passion. In this study, the Cronbach’s alpha coefficient was 0.898.

#### The Measurement of Psychological Capital

This survey used the PsyCap Questionnaire (PCQ-24) developed by Luthans et al. (2007b) and translated into Chinese by Li Chao-ping. The Chinese version of this tool had been applied to a previous study conducted among college students, which presented a good cross-cultural adaptation with high reliability and validity in China’s setting (Pan et al., 2015). The questionnaire contained six topics and 24 items that were assembled into four dimensions—self-efficacy, hope, toughness, and optimism. They were measured on a six-point Likert scale—1 implying *totally disagree* and 6 implying *totally agree*. Some items had been reversed. Higher scores indicated higher levels of PsyCap of medical students. In this study, the Cronbach’s alpha coefficient for the scale was 0.931.

#### The Measurement of Self-Esteem

A one-dimensional Self-esteem Scale containing a total of 10 items was developed by Rosenberger and was later translated and revised by Ji Yifu, Tian Yimei, and their colleagues (Rosenberg et al., 1978). A four-point Likert scale was used (1 = *completely inconsistent*, 2 = *sometimes consistent*, 3 = *often consistent*, 4 = *always consistent*). Some items had been reversed. A higher score reflected a higher self-esteem of medical students. In this study, the Cronbach’s alpha coefficient for the scale was 0.859.

#### The Measurement of Professional Identity

A one-dimensional instrument with six items suggested by Mael and Ashforth (1992) was used to measure professional identity, which had previously been widely used and has been proven to have good reliability and validity in a Chinese context (Peng et al., 2020). Participants were asked to provide responses on a five-point scale (1 implying *completely inconsistent* and 5 implying *always consistent*). Higher scores indicated higher professional identity. The Cronbach’s alpha coefficient for the scale was 0.842.

### Data Analysis

The main statistical methods included descriptive statistical analysis—to describe the demographic information of the participants and the status of learning passion—and Pearson correlation, which was tested to estimate the correlations between the two types of learning passion, PsyCap, and self-esteem. Hierarchical linear regression analysis was performed to test the associations and moderating and mediating effects of variables. In this study, *P* < 0.05 (two-tailed) was considered statistically significant. The previously mentioned analyses were conducted using SPSS 17.0 (IBM, BM SPSS Statistics for Windows).

## Results

### Demographic Information of the Sample

The demographic information of the sample included school, gender, provenience, academic year, gross annual household income, subjective academic performance and program, as presented in Table 1.

**TABLE 1:** Characteristics of respondents (*N* = 1,218).

Characteristics	Categories	*N* (%)
**School**		
	Chengde Medical College	223 (18.3)
	Qiqihar Medical College	277 (22.7)
	Mudanjiang Medical College	125 (10.3)
	Harbin Medical University	593 (48.7)
**Gender**		
	Male	264 (21.7)
	Female	953 (78.2)
	Unsure	1 (0.1)
**Origin of student**		
	Urban	672 (55.2)
	Non-urban	546 (44.8)
**Academic year**		
	First	736 (60.4)
	Second	194 (15.9)
	Third	93 (7.6)
	Fourth	146 (12.0)
	Fifth	49 (4.0)
Gross annual household income (RMB)	Less than 20,000 ¥	368 (30.2)
	20,001–50,000 ¥	380 (31.2)
	50,001–100,000 ¥	278 (22.8)
	100,001–200,000 ¥	150 (12.3)
	More than 200,000 ¥	37 (3.0)
	Unsure	5 (0.4)
Subjective academic performance	Superior level	140 (11.5)
	Upper middle level	383 (31.4)
	Medium level	414 (34.0)
	Middle and lower level	216 (17.7)
Program	Inferior level	65 (5.3)
	Clinical medicine	863 (70.9)
	Non-clinical medicine	355 (29.1)

*^¥^Legal tender symbol of the People’s Republic of China.*

### Status of Medical Students’ Learning Passion

Table 2 shows that the mean scores of harmonious and obsessive passion are (4.826 ± 0.90) and (4.114 ± 1.02), respectively. These results show that the overall level of learning passion of participants is relatively high, and the score of harmonious passion is higher than that of obsessive passion.

**TABLE 2:** Means, standard deviations (*SD*) and Pearson correlations of variables (*N* = 1,218).

Variables	Mean	*SD*	1	2	3	4
1. Self-esteem	2.986	0.409	1			
2. PsyCap	4.054	0.609	0.650**	1		
3. Harmonious learning passion	4.826	0.909	0.411**	0.594**	1	
4. Obsessive learning passion	4.114	1.023	0.236**	0.433**	0.641**	1

**p <0.01, Correlation is significant at the 0.01 level (2-tailed).

### Correlations Between Study Variables

The means, standard deviations, and Pearson correlation coefficients of variables are described in Table 2. The absolute value of the correlation coefficient is statistical significance, which indicates that each variable could be used in subsequent regression analyses to explore the association and mediating effects between them (Baron and Kenny, 1986).

### Multiple Linear Hierarchical Regression Models

Multiple linear regression analysis was used to test the relationship between PsyCap, self-esteem, and two types of learning passion, after eliminating the interference of the some demographic variables that may potential affect outcomes involving gender, academic year, program, and subjective academic performance, which was confirmed. Demographic information as control variables was variables that were statistically different in outcome variables in previous studies. Such variables are regarded as the control variables and are brought into Models 1, 3, and 7. As self-esteem was significantly positively associated with PsyCap (*M*_2_, β = 0.637, *P* < 0.01), H1 was confirmed; in addition, harmonious learning passion (*M*_5_, β = 0.400, *P* < 0.01) confirmed H4, and obsessive learning passion (*M*_9_, β = 0.234, *P* < 0.01) confirmed H5. Moreover, the PsyCap of participates was found to be significantly positively associated with harmonious learning passion (*M*_4_, β = 0.589, *P* < 0.01) and obsessive learning passion (*M*_8_, β = 0.436, *P* < 0.01). Therefore, H2 was supported, and H3 was confirmed. We relied on the four-step mediated regression approach recommended by Baron and Kenny (1986) and showed the results of the mediation analysis in Table 3. We found that PsyCap had a fully mediating effect on the relationship (*M*_6_, β = 0.578, *P* < 0.01) between self-esteem and harmonious learning passion; thus, H8 was confirmed. However, PsyCap had a suppression effect on the relationship between self-esteem and obsessive learning passion (*M*_10_, β = 0.490, *P* < 0.01). Therefore, H9 was confirmed in Table 3.

**TABLE 3:** Hierarchical linear regression analysis models (*N* = 1,218).

Variables	PsyCap		Harmonious learning passion				Obsessive learning passion			
	*M*_1_ (β)	*M*_2_ (β)	*M*_3_ (β)	*M*_4_ (β)	*M*_5_ (β)	*M*_6_ (β)	*M*_7_ (β)	*M*_8_ (β)	*M*_9_ (β)	*M*_10_ (β)
**Control variables**										
Gender	−0.069*	−0.052*	−0.066*	–0.026	−0.069*	–0.045	0.011	0.041	0.016	0.044
Academic year	−0.087**	−0.066**	−0.063*	0.017	−0.058*	–0.022	−0.061*	–0.027	–0.055	–0.023
Major	–0.047	–0.040	–0.035	0.002	–0.024	0.001	–0.050	–0.026	–0.044	–0.013
Subjective academic performance	−0.158**	−0.077**	−0.166**	0.070**	−0.127**	−0.091**	−0.149**	−0.077**	−0.114**	−0.077**
Annual household income	0.108**	0.048*	0.006	0.057**	–0.029	−0.059*	−0.062*	−0.107**	−0.085**	−0.113**
**Mediating variable**										
PsyCap				0.589**		0.578**		0.436**		0.490**
**Independent variable**										
Self-esteem		0.637**			0.400**	0.024			0.234**	−0.083*
*F*	9.365**	135.486**	6.818**	104.828**	44.040**	89.751**	6.342**	48.740**	16.327**	40.415**
*R* ^2^	0.043**	0.438**	0.030**	0.362**	0.190**	0.376**	0.028**	0.209**	0.081**	0.214**
Δ*R*^2^	0.043**	0.395**	0.030**	0.332**	0.156**	0.337**	0.028**	0.182**	0.054**	0.186**

*M_1_, M_3,_ M_7_, the influence of demographic variables on the PsyCap, harmonious learning passion and obsessive learning passion; M_2_, the influence of self-esteem on the PsyCap; M_4,_ M_8_, the influence of the PsyCap on harmonious learning passion and obsessive learning passion; M_5,_ M_9_, the influence of self-esteem on harmonious learning passion and obsessive learning passion; M_6,_ M_10_, the influence of self-esteem and PsyCap on harmonious learning passion and obsessive learning passion.*

*p < 0.05, **P < 0.01.

### Multiple Linear Regression Analysis of Moderation

**FIGURE 2:**
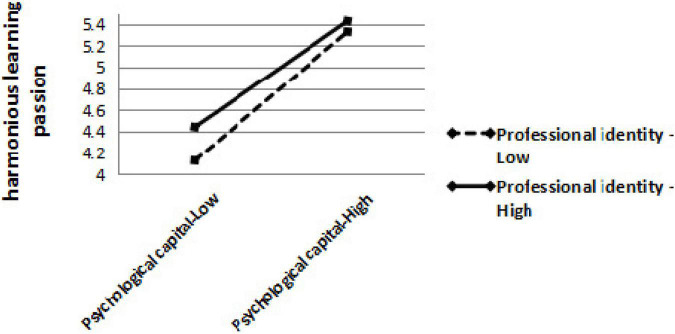
Moderator effect of professional identity on the relationship between PsyCap and harmonious learning passion.

**FIGURE 3:**
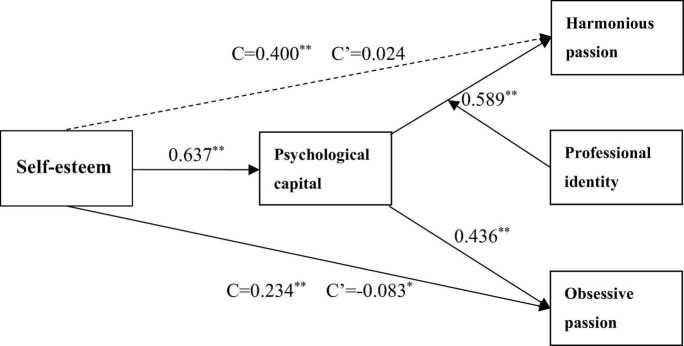
Modified model and standardized path coefficients. C is the total effect of self-esteem on harmonious passion and obsessive passion. C’ is the direct effect of self-esteem on harmonious passion and obsessive passion. *p < 0.05, **p < 0.01, Correlation is significant at the 0.01 level (2-tailed).

When the moderating effect was verified, according to the recommendations by Aiken and West (1991), the data were normalized (subtracted its average value) in Table 4. This study shows that professional identity significantly moderated the correlation between PsyCap and harmonious learning passion (β = −0.554, *P* < 0.01), rather than obsessive learning passion (*P* > 0.05); therefore, H6 was confirmed, and H7 was refused. To further demonstrate the trend of professional identity’s moderating effect and avoid a collinearity problem referring to the high correlation between independent variables and interaction term—after the data were centralized—we drew a diagram of the moderating role, as shown in Figure 2. Based on a series of findings in our study, we revised the relational model in Figure 3. The slope of low professional identity was higher than that of high professional identity, and the slope of low professional identity was more inclined. Compared with low professional identity, high professional identity weakened the influence of PsyCap on harmonious learning passion.

**TABLE 4:** Moderated regression analysis (*N* = 1,218).

Variable	Harmonious learning passion		Obsessive learning passion	
	*B*	*P*	*B*	*P*
**Cause variable**	0.885	<0.01	0.370	<0.01
PsyCap	0.885	<0.01	0.370	<0.01
**Moderator**				
Professional identity	0.476	<0.01	0.185	>0.05
**Interaction**				
PsyCap*Professional identity	−0.554	<0.01	0.017	>0.05
*R* ^2^	0.367	<0.01	0.223	<0.01
Δ*R*^2^	0.367	<0.01	0.223	<0.01
*F*	213.529	<0.01	105.020	<0.01

Professional identity-interaction, PsyCap* professional identity.

## Discussion

### Status of Medical Students’ Learning Passion

The overall mean score (standard deviation) of Chinese medical students’ harmonious passion and obsessive passion were 4.826 (0.90) points and 4.114 (1.02) points, respectively. Participants reported high scores of both types of learning passion, indicating that medical students maintained a high-degree enthusiasm for learning while exhibiting a passive emotional experience and even reluctant incentives (Zylan, 2011). This study indicates that, in medical students’ professional learning motivation, the level of harmonious learning passion—characterized by autonomous internalization—is higher than that of obsessive learning passion—characterized by compulsory internalization. By driving instrumental orientation, medical students’ learning engagement and passion are motivations for maintaining their subsequent profession’s livelihood and social status rather than professional calling (Zhang et al., 2020). Recently, medical practice and education have been emphasizing on motivation to stem from professional spirit, instead of livelihood. Thus, our findings suggest that medical educators and managers need to boost students’ learning passion because it would be a significantly meaningful measure for innovating the development pattern of medical education.

### Association Between Self-Esteem and Learning Passion in Medical Students: Mediating Effect of Psychological Capital

The results showed that self-esteem and PsyCap positively predicted the two types of learning passion among medical students. Self-esteem can directly predict obsessive learning passion and not directly predict harmonious learning passion among Chinese medical students. In China, *Mianzi* (face) is regarded as the recognition by others of an individual’s social standing and position, which is the most prominent Chinese cultural characteristics that have strong implications for Chinese people’s thinking, behavior and decision making process (Buckley et al., 2006). Influenced by the *Mianzi* culture, medical students think that it is vital to protect a their *Mianzi* or dignity and prestige, and force themselves to learn more responsive to the expectations from parents and teachers. Therefore, *Mianzi* culture can help us understand the current result. Owing to the *Mianzi* culture is conducive to driving the external learning motivation rather than self-growth-driven learning motivation, thereby self-esteem can directly affect obsessive learning passion and not directly affect harmonious learning passion for Chinese medical students.

PsyCap had a full mediating effect on the relationship between self-esteem and harmonious learning passion while suppressing the relationship between self-esteem and obsessive learning passion; this finding was inconsistent with a previous study (Larson and Luthans, 2006).

Self-esteem is a kind of overall self-evaluation with a self-affirmative tendency (Rosenberg, 1965b) that has a remarkable correlation with the increasingly positive cognition and behaviors of medical students—to the extent that individual it is contingent on a relatively stable source. Individuals with high self-esteem are better at understanding and taking advantage of their strengths and avoiding their weaknesses comprehensively (Wang et al., 2020). Thus, they are not affected by negative effects or setbacks during their daily study and maintain a positive outlook toward every situation, irrespective of the outcome (Chin, 2014). Additionally, medical students with high-level hope and high-degree optimism toward the future tend to have a greater propensity to being autonomous in learning regulations. They are more likely to devote to their passionate learning style with adequate openness to aim for better academic performance and clinical skills. Furthermore, students with higher PsyCap also exhibit optimistic completion of learning activities; they are emotionally driven to pursue their goals, resulting in a better balance between learning and other life activities. Therefore, students with positive self-evaluations and self-worth are prone to trigger a process of autonomous internalization to begin and maintain. Further, they engage in learning activities to reach the desired academic goals through the positive integration of individual psychological resources—self-efficacy, hope, optimism, and resilience (Luthans et al., 2007b).

Moreover, this study proves that students’ self-esteem and obsessive passion are negatively related, after controlling for the effects of PsyCap. The differences due to PsyCap would mask the effects of students’ self-esteem on obsessive passion, creating the observed suppression effect. For those students with obsessive passion, their actions have often been energized, coerced, or seduced by desired environmental conditions, involving parental expectations or their volition. Medical students with a positive PsyCap involving psychological resilience are likely to be skilled in coping with intrapersonal and/or interpersonal pressure owing to external support and high self-esteem. This tends to launch a self-driven process of controlled internalization of students’ learning into self-identity. Furthermore, they tend to exhibit reasonable control in the way they achieve their learning goals by properly balancing learning behaviors and other activities, which further augment their enthusiasm toward learning behavior. A previous study had indicated that students with high PsyCap generally have a stronger ability to alleviate learning pressure and decrease learning burnout in academic circumstances (Cheung et al., 2011). Analogously, medical students with high levels of stable self-esteem tend to exhibit greater psychological resilience and can actively regulate emotional states when they are facing frustration, academic stress, or other difficulties. They always maintain a higher level of passionate learning or force themselves to complete the learning objectives. Additionally, students with high self-esteem and resilience are prone to a greater persistence on tasks after a failure, although the persistence is non-productive or forced. Overall, self-esteem, as a psychological resource to increase one’s environmental adaptability (Steffenhagen, 1990), tends to enhance an individual’s ability to resist pressure and mental toughness. This helps one in effectively adjusting emotional states and pressure during the process of learning and living, which further results in a greater learning passion. Therefore, this study contributes to the existing literature by providing educators and policymakers with methods to boost students’ learning passion by strengthening PsyCap interventions and maintaining stable and high self-esteem.

### Association Between Psychological Capital and Learning Passion in Medical Students: Moderating Effect of Professional Identity

This study demonstrates how professional identity interactively shaped the relationship between PsyCap and harmonious learning passion rather than obsessive learning passion. It shows differences in how the internalization of a learning activity into a student’s identity occurs. Further, it demonstrates how the degree of identity internalization is greater for those with obsessive passion compared to those with harmonious learning passion. As PsyCap and professional identity have complementary effects on the two types of learning passion, this finding contributes to initially empirical support for responding to recent contentions that professional identity may have interactive effects. That is, students with low-degree PsyCap will boast greater harmonious learning passion among those with high professional identity more than those with low professional identity. Professional identity is an individual’s subjective feeling toward the specialty in daily life and represents the degree of consistency and balance between the individual and the profession (Olesen, 2001). In a medical education context, positively emotional identity tends to enable the individual to obtain better psychological identification and emotional pleasure and satisfaction. This inner emotional pleasure can directly cause positive motivations and lead to explicit behavioral outcomes, which prompts a sustainable professional identity (Sun et al., 2017). A theory of social identity pointed out that the most basic motivation of identity is the enhancement of individual self-esteem and that identity can promote a positive self-concept (Tajfel, 2003). Medical students with high professional identity have a strong sense of identity with the medical major and are more likely to be willing to love medical work and career; for these reasons, they may be directly inspired to devote more energy to learning. Thus, for medical students with low professional identity, the effect of their PsyCap on harmonious learning passion is greater. Medical students with obsessive learning passion also work on learning activities; however, they often feel compelled to engage in those driven by strategic contingencies (e.g., responding to parental expectations, pressures in finding jobs, environmental factors) that may control them.

### Limitations

Although this study makes significant discoveries, the following limitations cannot be ignored. First, as the samples were extracted from only four medical universities in Northern China, our results may be biased because of institutional and geographical influence and may not completely represent all Chinese medical students. Second, we used several foreign measuring tools that ignored issues of cross-cultural adaptability, such as professional identity, which offers the scope for further academic attention in the future. Third, the data were collected from the self-report of students through an online survey, which may have led to potential response bias because of social desirability or unsure effect. Furthermore, as a network survey, it could only indicate the situation at one point in time. Fourth, because this is a cross-sectional study, it could not determine the causal relationship between variables. Finally, further research is still needed to test whether the results are appropriate in different cultural contexts or regions.

## Conclusion

The results of the study showed that medical students with high self-esteem present greater PsyCap, and they strengthened two types of passion for learning. Moreover, this research demonstrated that medical students’ professional identity and PsyCap determined a harmonious learning passion through a significant interaction effect, rather than obsessive passion. In other words, the positive effect of PsyCap on harmonious learning passion is more remarkable among medical students with lower professional identity. Therefore, this study provides an insight into facilitating a reform in medical education. Further, it acts as a practical guide for educators and policymakers in the medical education industry to inspire the learning enthusiasm of medical students and enhance the quality of medical staff training. These goals can be attained by using advanced intervention measures based on positive psychology, such as professional identity, PsyCap, and leaning passion.

## Data Availability Statement

The raw data supporting the conclusions of this article will be made available by the authors, without undue reservation.

## Ethics Statement

The research was reviewed and approved by the Ethics Committee of the Institutional Review Board of Harbin Medical University (ECHMU). Owing to the anonymous survey approach, written informed consent could not be obtained. However, electronic informed consent for the research was approved by the ECHMU and obtained from each participate.

## Author Contributions

TS and D-PC conceived and designed the study. S-EZ and S-AG drafted the manuscript. JT, Q-LL, MZ, X-HW, J-YZ, and L-BY collected the data and controlled quality. M-SW and S-EZ conducted the data analyses. All authors contributed to publishing the final manuscript, read, and approved the final manuscript.

## Conflict of Interest

The authors declare that the research was conducted in the absence of any commercial or financial relationships that could be construed as a potential conflict of interest.

## Publisher’s Note

All claims expressed in this article are solely those of the authors and do not necessarily represent those of their affiliated organizations, or those of the publisher, the editors and the reviewers. Any product that may be evaluated in this article, or claim that may be made by its manufacturer, is not guaranteed or endorsed by the publisher.

## Footnotes

1
https://www.wenjuan.com/
